# Diagnostic accuracy of a liquid chromatography-tandem mass spectrometry assay in small hair samples for rifampin-resistant tuberculosis drug concentrations in a routine care setting

**DOI:** 10.1186/s12879-020-05738-5

**Published:** 2021-01-22

**Authors:** John Metcalfe, Peter Bacchetti, Ali Esmail, Andrew Reckers, David Aguilar, Anita Wen, Shu Huo, Winnie R. Muyindike, Judith A. Hahn, Keertan Dheda, Monica Gandhi, Roy Gerona

**Affiliations:** 1 Division of Pulmonary and Critical Care Medicine, Zuckerberg San Francisco General Hospital and Trauma Center, University of California, San Francisco, 1001 Potrero Avenue, Rm 5K1, San Francisco, CA 94110-0111 USA; 2 Department of Epidemiology and Biostatistics, UCSF, San Francisco, CA USA; 3 Lung Infection and Immunity Unit, Division of Pulmonology, University of Cape Town, Cape Town, South Africa; 4 Maternal-Fetal Medicine Division, Department of Obstetrics, Gynecology and Reproductive Sciences, University of California, San Francisco, San Francisco, CA USA; 5 Mbarara University of Science and Technology, Mbarara, Uganda; 6 Division of HIV, Infectious Diseases and Global Medicine, Department of Medicine, UCSF, San Francisco, CA USA

**Keywords:** LC-MS/MS, Hair, Multidrug-resistant tuberculosis

## Abstract

**Background:**

Treatment monitoring of drug-resistant tuberculosis (DR-TB) in resource-limited settings is challenging. We developed a multi-analyte assay for eleven anti-TB drugs in small hair samples as an objective metric of drug exposure.

**Methods:**

Small hair samples were collected from participants at various timepoints during directly observed RR-TB treatment at an inpatient tertiary referral facility in South Africa (DR-TB cohort). We assessed qualitative determination (i.e., detection above limit of detection) of bedaquiline, linezolid, clofazimine, pretomanid, levofloxacin, moxifloxacin, pyrazinamide, isoniazid, ethambutol, ethionamide, and prothionamide in an LC-MS/MS index panel assay against a reference standard of inpatient treatment records. Because treatment regimens prior to hospitalization were not available, we also analyzed specificity (for all drugs except isoniazid) using an external cohort of HIV-positive patients treated for latent TB infection with daily isoniazid (HIV/LTBI cohort) in Uganda.

**Results:**

Among the 57 DR-TB patients (58% with pre-XDR/XDR-TB; 70% HIV-positive) contributing analyzable hair samples, the sensitivity of the investigational assay was 94% or higher for all drugs except ethionamide (58.5, 95% confidence interval [CI], 40.7–99.9). Assay specificity was low across all tested analytes within the DR-TB cohort; conversely, assay specificity was 100% for all drugs in the HIV/LTBI cohort.

**Conclusions:**

Hair drug concentrations reflect long-term exposure, and multiple successive regimens commonly employed in DR-TB treatment may result in apparent false-positive qualitative and falsely elevated quantitative hair drug levels when prior treatment histories within the hair growth window are not known.

## Background

An estimated 1.5 million people globally have rifampin-resistant tuberculosis (RR-TB). Treatment of RR-TB in the setting of HIV co-infection is complicated by high pill burden [1], overlapping drug toxicities [2], poor drug absorption [3, 4], and high mortality [5]. Because of non-invasive collection, easy storage, and long-term detection window, hair concentrations of anti-TB medications represent an important objective determination of patient adherence and a measure of individual pharmacodynamics.

Determination of drug concentrations in small hair samples has long informed forensic investigation and environmental exposure assessment, where the liquid chromatography- tandem mass spectrometry (LC-MS/MS) measures are considered highly sensitive and specific. Assuming a scalp hair growth rate of approximately 1 cm per month, historic drug exposure can be ascertained via segmental analysis, determining drug levels in various sections of hair with successive distance from the root [6]. Stringent in vitro validation procedures provide international guidance for method development [7], though real-world assessments occasionally lead to false-positive (e.g., external contamination) or false-negative (e.g., cosmetic hair treatments) results.

Recently, we developed and validated a multi-drug assay panel for measuring eleven anti-tuberculosis drug concentrations in small hair samples [8, 9]. Here, we report a clinical diagnostic accuracy study of qualitative results for individual drugs (as determined by detection above the assay’s limit of detection (LOD)) as measured against a gold standard of inpatient treatment administration records in a routine care setting in South Africa. In a *post-hoc* assessment, we validated assay specificity within an external cohort of patients receiving isoniazid alone for latent TB infection (LTBI) where ingestion of drugs used to treat RR-TB would be highly improbable.

## Methods

### Study population and sample collection

The primary study cohort of adult (aged ≥ 18 years) patients with MDR-, pre-XDR, and XDR-TB was recruited as a convenience sample from July 12, 2016 to December 6, 2017 at Brooklyn Chest Hospital (BCH), an inpatient referral facility for drug-resistant TB in Cape Town, Western Cape, South Africa. During the study period, pre-XDR and XDR-TB patients were treated with 24 weeks of bedaquiline within an optimized, individualized background regimen that could include levofloxacin, linezolid, and/or clofazimine. Inpatient treatment was administered by directly observed treatment (DOT); treatment administered prior to the hospital stay was administered according to programmatic standards at each peripheral clinic, which may or may not have included DOT. Small hair samples were collected as previously described [8] at a single time point during the patient’s final treatment regimen (i.e., prior to last known treatment outcome). Patients with cosmetically treated hair (including bleaching) or whose scalp hair was less than or equal to 2 mm in length were excluded.

A secondary study cohort of adult HIV-positive patients on ART with LTBI was enrolled in a longitudinal cohort study at the Mbarara Regional Referral Hospital in Uganda. The participants in this study had LTBI confirmed by tuberculin skin testing (≥5 mm induration) with active and prior TB and TB drug exposure ruled out; all participants were given daily self-administered 300 mg INH. This cohort was chosen for a specificity analysis of our multi-analyte assay given the low likelihood of exposure to second-line anti-TB medications but high HIV prevalence. In this secondary cohort, hair was collected after 3 and 6 months of INH and analyzed at both timepoints when available.

Small hair samples were collected using previously described methods [10]. Briefly, from all participants with scalp hair and who consented for hair collection, 20–30 strands of hair were cut from the occipital region. The distal end of the hair sample was marked with a small piece of tape to denote directionality, and the hair was stored in aluminum foil at room temperature. Each participant provided written informed consent, and ethical approval was obtained from the University of Cape Town Human Research Ethics Committee (187/2016), the Mbarara University of Science and Technology Research Ethics Committee (11/10–16), and the University of California, San Francisco (UCSF) Human Research Protection Program (14–14,609). All hair samples were analyzed at the UCSF TB Hair Analysis Laboratory.

### Investigational LC-MS/MS assay

We analyzed the samples using our validated LC-MS/MS method for the simultaneous quantitation of eleven MDR-TB drugs in small hair samples [11]. Briefly, hair strands (~ 20–30 cut to 3 cm and weighed to 2 mg) were pulverized and extracted with methanol. The hair extract was reconstituted to water with 1% formic acid before injection into the Agilent LC 1260 (Agilent Technologies, Sta. Clara, CA) attached to an AB Sciex API 5500 mass spectrometer (AB Sciex, Foster City, CA). The analytes were separated by gradient elution on a Phenomenex Synergi Polar RP column (2.1 × 100, 2.5 μm particle size, Phenomenex, Torrance, CA) using water with 1% formic acid as mobile phase A (MPA) and acetonitrile with 0.1% formic acid as mobile phase B (MPB). Ionization of each analyte in the mass spectrometer was achieved using electrospray ionization (ESI) in positive polarity, and mass scanning was performed via multiple reaction monitoring (MRM). Quantification of each analyte was performed by isotope dilution method using deuterated, ^15^N- or ^13^C-labeled isotopologue of each drug standard. Data analysis was done using AB Sciex Analyst 1.6 and AB Sciex MultiQuant 2.1 (AB Sciex, Foster City, CA) software packages. Diagnostic specificity was also monitored during method validation using hair samples from laboratory members who have not taken any of the drugs in the panel [9].

### Statistical analysis

The primary objective of the study was to determine the sensitivity and specificity of the investigational assay analyzing qualitative hair concentrations of INH, pyrazinamide (PZA), ethambutol (EMB), levofloxacin (LFX), moxifloxacin (MFX), linezolid (LZD), clofazimine (CFZ), bedaquiline (BDQ), pretomanid (PTM), ethionamide (ETH), and prothionamide (PTH), against a reference standard of known drug administration, according to data abstracted from inpatient treatment records. In the primary analyses, the reference standards for sensitivity and specificity were considered differently. For sensitivity, the reference standard was considered ‘positive’ if the drug was taken for at least 14 days during the hair growth window. The hair growth window was defined as the interval from 94 to 5 days prior to hair collection. Due to prolonged half-lives, the hair growth window was defined as an interval beginning 800 and 420 days prior to hair collection for BDQ and CFZ, respectively (i.e., a time period encompassing roughly five half-lives). For specificity, the reference standard was considered ‘negative’ if the drug is not taken for any days in the previous 124 days prior to and including the day of hair collection. Because of the long half-lives of BDQ and CFZ, the reference standard was ‘negative’ if there was no known history of past use of these drugs at any time. Specificity was also calculated separately using a secondary, external cohort described above. Investigational assay analysis was performed independently of reference standard treatment data. The binomial exact method was used to calculate 95% confidence intervals using Stata 14.2 [12].

## Results

### Participants

**Fig. 1:**
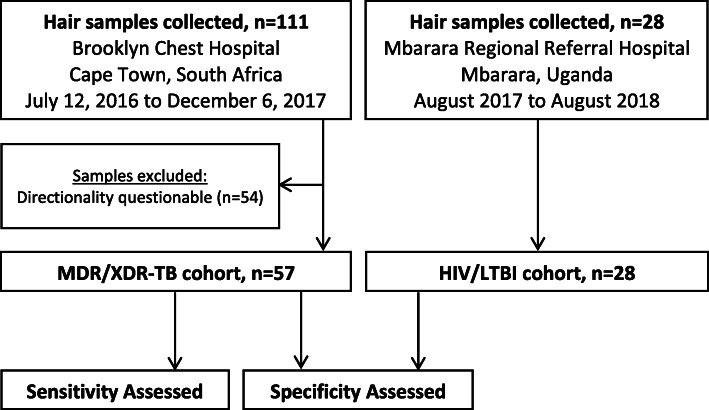
Participant Enrollment and Testing

A total of 111 participants with DR-TB were enrolled from July 12, 2016 to December 6, 2017 (Fig. 1). Among 54 participants (49%), directionality of the hair sample (i.e., differentiation of distal and proximal hair segments) could not be reliably determined. Thus, 57 participants with MDR/XDR-TB were eligible for inclusion in the study. These participants were predominantly female (98%, *n*=56/57) and HIV-positive (70%, *n*=40/57; 95% (*n*=38/40) on ART). A broad spectrum of phenotypic resistance patterns was represented, and participants had been on treatment for a median of 144 days (interquartile range (IQR) 50–337 days) prior to hair sampling (Table 1). The LTBI samples (*n*=28, from 19 persons) were collected from 42% females; all were HIV-positive and all were on ART. The total number of patients approached but declining to participate was not recorded.

**Table 1:** Demographic and clinical characteristics of participants at enrollment

Characteristic	MDR/XDR-TB cohort (primary)
Female sex — no./total no. (%)	56/57 (98%)
Median age (range) — yr	33 (22–58)
Time from most recent diagnosis to small hair sample collection (median, IQR) — days	144 (50–337)
Drug-resistance status — no./total no. (%)	
Pre-XDR/XDR-TB	36/57 (63%)
MDR-TB	16/57 (28%)
Poly-resistance	1/57 (2%)
RR-TB	4/57 (7%)
Treatment Outcome — no./total no. (%)	
Loss to follow-up	14/57 (25%)
Treatment failure	6/57 (11%)
Relapse	3/57 (5%)
Continuing Treatment	18/57 (32%)
Cure	15/57 (26%)
Complete	1/57 (2%)

### Investigational assay versus treatment administration data in primary and secondary cohort

The sensitivities of the investigational assay for the detection of administered drugs were 93.9% or higher for all drugs except ETH (Table 2). Specificity, however, was high for only a few drugs, and several had upper 95% confidence bounds below 50%. In contrast, specificity as ascertained within the secondary HIV/LTBI cohort was 100% (95% CI, 88–100%) for all analytes excepting INH, for which specificity could not be ascertained due to INH treatment among all participants. Within the primary cohort, the distributions of above-LOD drug concentrations were substantially lower among those without known treatment histories (i.e., false-positives) relative to those with confirmed treatment histories (i.e., true positives), with the first quartile among true positives higher than the third quartile among false positives for PZA, EMB, LFX, MFX, LZD, CFZ, and BDQ (Table 2).

**Table 2:** Sensitivity and specificity of the investigational assay, with treatment history as the reference standard

Drug	Sensitivity^**a**^		Specificity^**b**^		Drug concentration (ng/mg)	
					True positives	False positives
	*No. detected/no. taking drugs*	*% (95% CI)*	*No. not detected/no. not taking drugs*	*% (95% CI)*	*Median (IQR)*	*Median (IQR)*
**Isoniazid**	31/33	93.9 (79.8–99.3)	12/20	60 (36.1–80.9)	0.99 (0.32–2.21)	0.33 (0.25–0.46)
**Pyrazinamide**	54/54	100 (93.3–100)	0/2	0 (0–84.2)	9.84 (4.52–18.54)	0.26 (0.22–0.38)
**Ethambutol**	45/45	100 (92.1–100)	0/10	0 (0–30.8)	0.92 (0.43–1.53)	0.05 (0.02–0.35)
**Levofloxacin**	30/30	100 (88.4–100)	2/26	7.7 (0.9–25.1)	26.46 (11.53–48.08)	0.63 (0.32–1.06)
**Moxifloxacin**	41/41	100 (91.4–100)	0/13	0 (0–26.5)	13.81 (6.23–18.32)	2.04 (0.46–4.91)
**Linezolid**	22/23	95.6 (78.1–99.9)	26/32	81.3 (64.6–92.8)	9.12 (4.20–17.66)	0.16 (0.14–0.43)
**Clofazimine**	34/34	100 (89.7–100)	2/22	9.1 (1.1–29.2)	2.98 (1.39–4.59)	0.20 (0.05–0.67)
**Bedaquiline**	27/27	100 (87.2–100)	7/28	25 (10.7–44.9)	0.90 (0.71–1.46)	0.21 (0.10–0.35)
**Pretomanid**	1/1	100 (2.5–100)	55/56	98.2 (90.4–100)	-^c^	–
**Ethionamide**	20/34	58.5 (40.7–99.9)	20/20	100 (83.2–100)	–	–

^a^ For sensitivity, the reference standard was considered ‘positive’ if the drug was taken for at least 14 days during the hair growth window

^b^ For specificity, the reference standard was considered ‘negative’ if the drug is not taken for any days in the previous 124 days prior to and including the day of hair collection. For BDQ and CFZ, the reference standard was ‘negative’ if there was no known history of past use of the drug at any time

^c^ Not enough data points for comparison

## Discussion

Measuring medication concentrations in small hair samples can provide a long-term metric of adherence and exposure. We assessed the diagnostic accuracy of a novel multi-analyte hair assay for eleven anti-TB drugs used in RR-TB treatment regimens. In a primary cohort of patients with extensive drug resistance and multiple prior TB regimens, our assay identified drug in hair with high sensitivity but apparent poor specificity. In order to determine the etiology of the high proportion of “false-positives” in the primary cohort, we next assessed specificity in a cohort of patients with a very low likelihood of exposure to anti-TB drugs other than INH. We determined that apparent false-positive assays in the primary cohort were most likely due to prior undocumented treatment histories. Research studies or clinicians utilizing hair PK assays for treatment monitoring in similar populations should note this possibility in interpreting assay results.

Our observation can be better understood by considering what is known about how xenobiotics accumulate in hair. Xenobiotics are thought to incorporate into hair (a pure collagen matrix in mammals) through passive diffusion into the growing hair follicle via surrounding arterial capillaries, through deposition via sweat and sebum after emergence from the scalp, and through external contamination (e.g., through physical contact). For a given absolute drug exposure and hair growth rate, incorporation of specific drugs into hair is a function of basicity, lipophilicity, and melanin content of hair, all of which increase hair drug concentrations. As an objective treatment monitoring tool, the major practical advantage of the hair biomatrix relative to blood or urine is the extended surveillance window for drug exposure over prior weeks to months, rather than hours to days [6]. A heuristic for estimating the period of drug exposure assumes a constant 1 cm/month hair growth rate (e.g., a 2-cm segment of hair corresponds to a 2 month period of drug exposure). However, a broader band of positivity from single doses of drugs have been noted in the forensic literature [13–15]. This variability in the area over which incorporated drug can be distributed in the hair shaft can be due to actual rate of hair growth (range in healthy subjects of at least 0.3 to 1.8 cm/mo) [15], rate of axial distribution of drug, and a number of other biologic characteristics.

We found a high sensitivity of our hair assay for all the DR-TB drugs except for ethionamide (most likely due to the poor incorporation of ethionamide in hair, as previously noted) [14]. Although patient management during the duration of our study in South Africa included inpatient treatment of drug-resistant TB, first- and second-line regimens are often started prior to inpatient treatment referral. In addition, programmatic diagnosis of drug-resistant TB can be delayed by to up to 2 months, during which time patients are often treated with first-line regimens. A high specificity of our assay is supported by findings in our *post-hoc* secondary cohort, as well as by the finding of substantially lower drug concentrations among individuals in our primary cohort without confirmed drug exposure in the hair growth window. In theory, this indicates that prior treatment regimens not otherwise discernable could be objectively delineated through use of our panel assay. Nevertheless, use of our assay in programmatic settings or in research settings involving DR-TB retreatment will have to take account the possibility of prior successive TB treatment regimens in assessing hair drug concentrations.

An important limitation of our study is that initial sample collection failed to adequately delineate the proximal from the distal end of the hair thatch; this was later corrected, and these earlier samples were not used in our analyses. Additional measures to verify correct labeling of small hair samples, or, alternatively, analyzing the entire hair sample and standardizing by weight, is recommended. Second, we assessed the diagnostic accuracy of individual anti-TB drug concentrations above their limits of detection qualitatively against an inpatient treatment record gold standard. Although inpatient treatment records represent a reasonable reference standard, they may imperfectly represent true drug ingestion. Third, we designed a post hoc analysis to examine an unanticipated decrement in assay specificity for many drugs. However, we consider this as a co-publication of two separate sequential studies rather than as an unplanned subgroup analysis, and thus not subject to the dangers and limitations typically ascribed to the latter [16].

## Conclusion

In conclusion, we assessed the diagnostic accuracy of an 11-drug RR-TB panel assay to ascertain drug exposure in small hair samples in a routine care setting. Since hair concentrations reflect long-term exposure, use of prior medications may be reflected in hair even after discontinuation. Among patients with drug-resistant TB, failure to account for these prior regimens may result in apparent false-positive qualitative and falsely elevated quantitative hair drug levels. Additional investigations examining the quantitative association of medication concentrations in small hair samples with RR-TB treatment outcomes are ongoing.

## Data Availability

All data generated or analysed during this study are available from the corresponding author on reasonable request.

## Abbreviations

DR-TB: Drug-resistant tuberculosis
LC-MS/MS: Liquid Chromatography with tandem mass spectrometry
LTBI: latent TB infection
MDR-TB: Multidrug-resistant tuberculosis
XDR-TB: Extensively drug-resistant tuberculosis
RR-TB: Rifampin-resistant tuberculosis
LOD: Limit of detection
DOT: Directly observed treatment
ART: Antiretroviral therapy
INH: Isoniazid
MPA: Mobile phase A
MPB: Mobile phase B
ESI: Electrospray ionization
MRM: Multiple reaction monitoring
PZA: Pyrazinamide
EMB: Ethambutol
LFX: Levofloxacin
MFX: Moxifloxacin
LZD: Linezolid
CFZ: Clofazimine
BDQ: Bedaquiline
PTM: Pretomanid
ETH: Ethionamide
PTH: Prothionamide
